# Vasculogenic Mimicry Occurs at Low Levels in Primary and Recurrent Glioblastoma

**DOI:** 10.3390/cancers15153922

**Published:** 2023-08-01

**Authors:** Kelsey Maddison, Sam Faulkner, Moira C. Graves, Michael Fay, Nikola A. Bowden, Paul A. Tooney

**Affiliations:** 1 School of Biomedical Sciences and Pharmacy, The University of Newcastle, Callaghan, NSW 2308, Australia; kelsey.maddison@uon.edu.au (K.M.); sam.faulkner@newcastle.edu.au (S.F.); paul.tooney@newcastle.edu.au (P.A.T.); 2 Mark Hughes Foundation Centre for Brain Cancer Research, The University of Newcastle, Callaghan, NSW 2308, Australia; moira.graves@newcastle.edu.au (M.C.G.); michael.fay@newcastle.edu.au (M.F.); 3 Drug Repurposing and Medicines Research Program, Hunter Medical Research Institute, New Lambton Heights, NSW 2305, Australia; 4 School of Medicine and Public Health, The University of Newcastle, Callaghan, NSW 2308, Australia; 5 GenesisCare, Lake Macquarie Private Hospital, Gateshead, NSW 2290, Australia

**Keywords:** glioblastoma, vasculogenic mimicry, angiogenesis, prostate-specific membrane antigen, tumour recurrence, tumour progression

## Abstract

**Simple Summary:**

Glioblastomas are resistant to treatments targeting angiogenic blood vessel development. It is possible that glioblastoma cells are forming vessel-like structures, called vasculogenic mimicry (VM), and contributing to treatment resistance through this process. We aimed to quantify VM in primary and recurrent glioblastoma and to determine whether VM vessels express the pathological vessel marker prostate-specific membrane antigen (PSMA). We found that only a small proportion of vessels in glioblastoma were VM, and that these vessels did not express PSMA. However, the expression of PSMA was decreased in recurrent compared to primary tumours, as was the total vessel density. The potential of VM as a treatment target and its contribution to treatment resistance in glioblastoma require further investigation.

**Abstract:**

Vasculogenic mimicry (VM), the ability of tumour cells to form functional microvasculature without an endothelial lining, may contribute to anti-angiogenic treatment resistance in glioblastoma. We aimed to assess the extent of VM formation in primary and recurrent glioblastomas and to determine whether VM vessels also express prostate-specific membrane antigen (PSMA), a pathological vessel marker. Formalin-fixed paraffin-embedded tissue from 35 matched pairs of primary and recurrent glioblastoma was immunohistochemically labelled for PSMA and CD34 and stained with periodic acid–Schiff (PAS). Vascular structures were categorised as endothelial vessels (CD34+/PAS+) or VM (CD34−/PAS+). Most blood vessels in both primary and recurrent tumours were endothelial vessels, and these significantly decreased in recurrent tumours (*p* < 0.001). PSMA was expressed by endothelial vessels, and its expression was also decreased in recurrent tumours (*p* = 0.027). VM was observed in 42.86% of primary tumours and 28.57% of recurrent tumours. VM accounted for only a small proportion of the tumour vasculature and VM density did not differ between primary and recurrent tumours (*p* = 0.266). The functional contribution of VM and its potential as a treatment target in glioblastoma require further investigation.

## 1. Background

Glioblastoma is the most commonly occurring adult primary malignant brain tumour [1].

Despite an aggressive standard treatment approach of maximal safe surgical resection, radiation therapy, and temozolomide (TMZ) chemotherapy, the median survival time for glioblastoma is poor at 14.6 months [1,2]. Additionally, there are limited treatment options available upon disease progression, when resistance to the standard treatment occurs and a recurrent tumour develops. As glioblastomas are highly vascularised tumours that often contain distinct patterns of microvascular proliferation, angiogenesis has been an appealing target for treatment in recurrent tumours [3,4]. However, bevacizumab, a monoclonal antibody directed against vascular endothelial growth factor (VEGF), has not demonstrated a significant overall survival benefit [5,6,7]. Alternative mechanisms of vascularisation, including vasculogenic mimicry, may contribute to anti-angiogenic treatment resistance and compensate for insufficient angiogenic vessel formation.

Vasculogenic mimicry (VM) is the formation of a functional vascular network by tumour cells [8]. VM was first described in melanoma tissue as matrix-associated loops and networks that opened into hollow channels containing red blood cells, could be stained with periodic acid–Schiff (PAS), and lacked an endothelial cell lining [8]. The presence of these tumour-cell-dependent vascular networks has been associated with poor prognosis in melanoma, as well as tumour aggressiveness and invasiveness [8,9,10]. VM has been reported in primary glioblastoma tissue [11,12,13,14], where its presence has been demonstrated to be a predictor of poor prognosis [13,14]. However, the presence of VM in recurrent glioblastoma has not been thoroughly studied. 

Prostate-specific membrane antigen (PSMA) is a transmembrane glycoprotein expressed in the normal prostatic epithelium, renal tubules, and small intestine [15,16]. It is also expressed by the neovasculature of a number of solid tumours, including glioblastoma, but is not expressed by normal vasculature [15,16,17,18]. In the context of angiogenesis, PSMA acts downstream of matrix metalloproteinase-2 (MMP-2) to cleave laminin into pro-angiogenic fragments, promoting endothelial cell adhesion and migration [19]. VM-capable melanoma cells demonstrate increased expression of the laminin 5 γ2 chain and MMP-2 [9], both of which are also associated with VM formation in glioblastoma [20,21]. PSMA may also be expressed by glioblastoma cells [18], and whether PSMA is involved in VM as well as angiogenesis is unclear.

Since the extent to which VM occurs in recurrent glioblastoma has not previously been studied, we aimed to determine whether VM vessels were present in recurrent tumours and whether VM density differed between primary and recurrent glioblastoma. We also aimed to assess whether PSMA expression occurred in both angiogenic and VM vessels. 

## 2. Materials and Methods

### 2.1. Tissue Samples

This study was approved by the Human Research Ethics Committee of the University of Newcastle (H-2018-0007). Formalin-fixed paraffin-embedded (FFPE) tumour tissue from 35 cases of glioblastoma with matched primary and recurrent tumour tissue was sourced from the Mark Hughes Foundation Brain Cancer Biobank facilitated by the NSW Regional Biospecimen Services (The University of Newcastle). Glioblastoma was diagnosed histologically at the time of primary tumour resection by a Hunter Area Health Service pathologist according to either the 2007 or 2016 World Health Organisation Classification of Tumours of the Central Nervous System criteria. Clinical information available for the cohort included median age at diagnosis (years), patient sex, treatment after primary resection, IDH1 mutation status, MGMT promoter methylation status, recurrent tissue collection timepoint, and median overall survival (days) (Supplementary Table S1).

### 2.2. Immunohistochemistry

FFPE tissue was sliced into 4 µm sections and processed by the NSW Regional Biospecimen Services for automated IHC using the Ventana Discovery Ultra Staining System (Ventana Medical Systems, Tucson, AZ, USA). Sections were labelled using pre-diluted rabbit monoclonal PSMA (EP192; Ventana Medical Systems, Tucson, AZ, USA) and mouse monoclonal CD34 (QBEnd/10; Ventana Medical Systems, Tucson, AZ, USA) as the primary antibodies. Slides were loaded into the instrument and tissue sections were baked to the slides and deparaffinised. Antigen retrieval was performed at pH9/95 °C with an incubation time of 32 min. Blocking was performed before addition of the primary antibody, followed by incubation for 24 min at 36 °C. The appropriate secondary antibody was added (anti-rabbit for PSMA, anti-mouse for CD34; Ventana Medical Systems, Tucson, AZ, USA), followed by another 24 min incubation period. 3′,3′-diaminobenzadine (DAB) was used as the chromogen. Labelled slides were removed from the instrument. All slides were stained with PAS and counterstained with haematoxylin. Positive and negative control slides were included with each batch of slides labelled by the instrument. Slides were digitised at 400× absolute magnification using the Aperio Digital AT2 Pathology System (Leica Biosystems, Melbourne, VIC, Australia) and imported into the HALO^®^ image analysis platform (version 3.3, Indica Labs, Albuquerque, NM, USA) for analysis.

### 2.3. Tumour Vessel Quantification

CD34/PAS-labelled sections were used to assess glioblastoma vasculature. An annotation region was drawn around the perimeter of each tumour section in HALO^®^ to select the area to be analysed. Exclusion annotations were used to outline areas within the tumour that were to be excluded from vessel assessment, such as necrotic tissue. The tiled partitioner function was then used to place 10 boxes of 550 × 500 µm within the annotated region. Boxes were placed at least 550 µm apart and were not placed over any exclusion regions. Where more than 10 boxes were generated, the relevant number of boxes required to reduce to total number to 10 were removed at random by HALO^®^. The number of endothelial, VM, and “mosaic” vessels were counted within each of the 10 regions per section. Endothelial vessels were CD34+/PAS+, VM vessels were CD34−/PAS+, and vessels that were partially CD34+ and partially CD34− with a PAS+ basement membrane were considered mosaic vessels, as described in previous studies [13,20]. Densities for each vessel subset and total vessel density were calculated as the mean count/mm^2^.

### 2.4. PSMA Quantification

HALO^®^ image analysis platform was used to assess PSMA expression levels. The tumour perimeter was selected, and large areas of necrosis and adjacent normal tissue were manually excluded. Tissue classifier algorithms were trained to recognise PSMA+ labelling (i.e., DAB), tumour tissue, necrosis, and areas of the slide that did not contain tissue. The Area Quantification algorithm was trained and optimised to detect positive pixel intensities corresponding to PSMA labelling within the tumour. The algorithm was then used to quantify the total tissue area analysed for each tumour and the percentage of the tumour that was weakly, moderately, and strongly labelled for PSMA. PSMA expression values were calculated as an H-score for each tumour section using the equation: H-score = (1 × % of weakly labelled tissue) + (2 × % of moderately labelled tissue) + (3 × % of strongly labelled tissue). 

### 2.5. Statistical Analysis

Datasets were tested for normality using the Shapiro–Wilk test. Two-tailed Wilcoxon matched-pairs signed rank tests were used to assess median differences in total vessel density and densities of endothelial, VM, and mosaic vessel subsets between primary and recurrent tumour groups. A one-tailed Wilcoxon matched-pairs signed rank test was used to determine whether PSMA expression significantly decreased in the recurrent tumour group. Kendall’s tau-b correlations were run to determine the relationship between vessel densities, PSMA expression, and clinical characteristics. Kaplan–Meier survival analyses with log rank tests were used to determine whether there were differences in the overall survival (OS), progression-free survival (PFS), or post-progression survival distributions when tumours were grouped as being VM+/VM− or having above or below median PSMA expression. OS was defined as the time in days between the date of primary surgery and date of death, or last known follow-up for censored cases. PFS was defined as the time in days between the date of primary surgery and date of recurrent surgery, and post-progression survival was defined as the time in days from the date of recurrent surgery until date of death. Descriptive statistics are reported as median values for tests of group differences and survival analyses. Statistical analyses were performed using SPSS Statistics (version 28.0, IBM Corporation, Armonk, NY, USA) or GraphPad Prism (version 9.1.1, GraphPad Software, Boston, MA, USA). A *p*-value < 0.05 was considered statistically significant for all tests. 

## 3. Results

### 3.1. Clinical Characteristics

A total of 35 cases with matched primary and recurrent glioblastoma samples were included in this study. Twenty patients (57.14%) were male and 15 (42.86%) were female. The median age at diagnosis was 59 years (range 23–81 years) and the median OS time was 493 days (range 57–2531 days). Additional demographic and clinical information is summarised in Supplementary Table S1.

### 3.2. Vasculogenic Mimicry Is Present in Recurrent Glioblastoma

**Figure 1:**
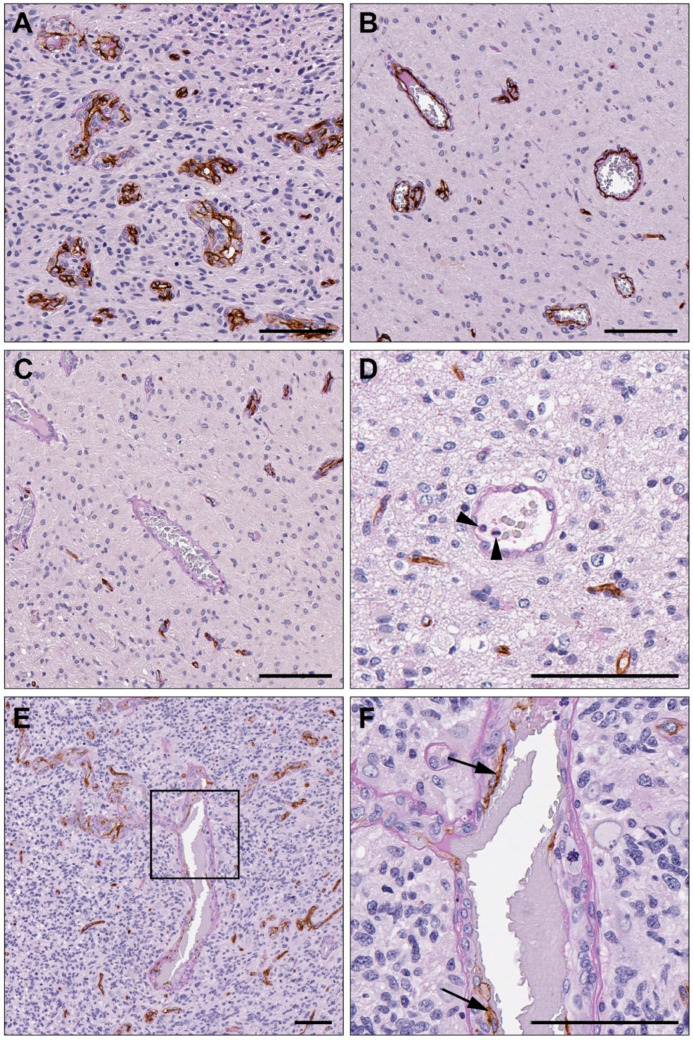
Representative images of blood vessel categories quantified within each tumour. Glioblastoma sections were labelled for CD34 by DAB IHC (brown) and stained with PAS (pink) to enable the identification of endothelial, VM, and mosaic vessels. CD34+/PAS+ endothelial vessels comprised the majority of vessels in all sections (**A**,**B**). CD34−/PAS+ VM vessels lacked an endothelial cell lining but were surrounded by a PAS+ vascular basement membrane (**C**,**D**). Red blood cells were frequently observed within the lumen of VM structures, and on some occasions, white blood cells (arrowheads) were also present (**D**). Mosaic vessels were partially CD34+ with a PAS+ basement membrane (**E**). The vessel in (**E**) is shown at a higher magnification in (**F**), where CD34 labelling (arrows) can be seen in only some segments of the vessel lining. Scale bars = 100 µm.

Immunohistochemical labelling for CD34 and staining with PAS were used to identify endothelial, VM, and mosaic vessels in glioblastoma tissue sections (Figure 1). Vessels with an endothelial cell lining were CD34+ with a PAS+ vascular basement membrane and accounted for the majority of the vasculature in all tumour sections. VM was defined as vascular structures without an endothelial lining and surrounded by a PAS+ basement membrane. These structures frequently contained red blood cells within their lumen, and in some cases, white blood cells were also observed (Figure 1). Vascular structures with PAS+ basement membranes that partially labelled for CD34 were categorised as mosaic vessels (Figure 1). All vessel categories were observed in both primary and recurrent tumours. Tumours were considered VM+ if they contained ≥1 VM (CD34−/PAS+) vessel in any of the 10 areas sampled for vessel counting. Fifteen primary tumours (42.86%) and 10 recurrent tumours (28.57%) were VM+.

### 3.3. Endothelial Vessel Density Decreases at Recurrence, While VM Density Does Not Change

**Figure 2:**
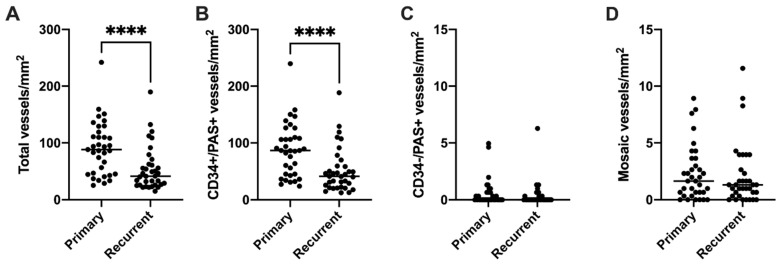
Total vessel density and vessel subset densities in primary compared to recurrent glioblastoma. Vessels in each glioblastoma section were categorised as endothelial (CD34+/PAS+), VM (CD34−/PAS+), or mosaic (partially CD34+). The mean number of vessels observed in ten sample areas per section was converted to number of vessels/mm^2^ and compared for each category between the primary and recurrent tumour groups. (**A**) There was a significant decrease in overall vessel density in recurrent compared to primary tumours (*z* = −3.80, *p* < 0.001). (**B**) The majority of tumour vasculature was made up of endothelial vessels, which also significantly decreased in density at recurrence compared to the primary tumour group (*z* = −3.759, *p* < 0.001). There was no change in VM vessel density (**C**) or mosaic vessel density (**D**) between groups (VM: *z* = −1.112, *p* = 0.266; mosaic: *z* = −0.661, *p* = 0.509). Median values are shown for each dataset. Note that VM and mosaic vessel data are plotted on a different scale for ease of visualisation. **** *p* < 0.0001.

After confirming that VM was present in recurrent glioblastoma, we determined whether any changes in vessel density occurred between primary and recurrent tumours. Data are expressed as median values, unless otherwise stated. The majority of cases (27/35) demonstrated a decrease in total vessel density at recurrence, and the decrease in total vessel density from primary (88.30 vessels/mm^2^) to recurrent (41.67 vessels/mm^2^) tumours was statistically significant (*z* = −3.80, *p* < 0.001; Figure 2A). Most vessels were CD34+/PAS+ endothelial vessels, and as such there was also a statistically significant decrease in endothelial vessel density in recurrent (41.34 vessels/mm^2^) compared to primary (86.98 vessels/mm^2^) glioblastomas (*z* = −3.759, *p* < 0.001; Figure 2B). The median density of CD34−/PAS+ VM vessels for both primary and recurrent tumours was 0.00 vessels/mm^2^, and there was therefore no difference in VM density between groups (*z* = −1.112, *p* = 0.266; Figure 2C). There was also no difference in median mosaic vessel density between the primary (1.65 vessels/mm^2^) and recurrent (1.32 vessels/mm^2^) groups (*z* = −0.661, *p* = 0.509; Figure 2D). Mean vessel counts are presented in Supplementary Table S2.

### 3.4. Presence of VM at Recurrence Is Associated with Shorter Post-Progression Survival

**Figure 3:**
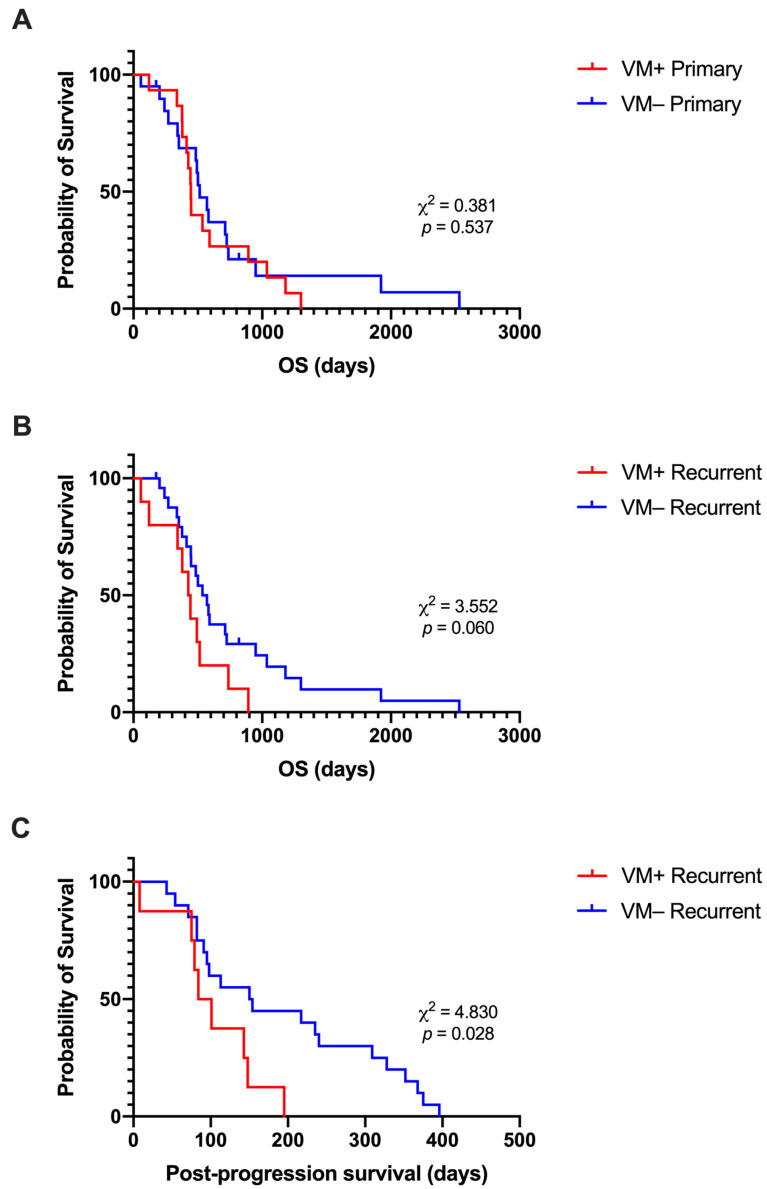
Kaplan–Meier plots of overall and post-progression survival distributions for VM+ and VM− glioblastoma. There was no significant difference in OS time when tumours were split into VM+ and VM− groups at either the (**A**) primary (χ^2^ (1) = 0.381, *p* = 0.537) or (**B**) recurrent (χ^2^ (1) = 3.552, *p* = 0.060) timepoint. (**C**) Post-progression survival was significantly longer for cases that were VM− at recurrence compared to those that were VM+ (χ^2^ (1) = 4.830, *p* = 0.028).

Kaplan–Meier survival analyses were conducted to compare the survival distributions of VM+ vs. VM− tumours to determine whether the presence of VM in the primary or recurrent tumour had an effect on OS, and whether the presence of VM at recurrence had an effect on post-progression survival. Patients with VM− primary tumours (*n* = 20) had a median OS time of 514 days, which was longer than the median OS time of 445 days for patients with VM+ tumours (*n* = 15). However, the survival distributions of the two groups were not significantly different (χ^2^ (1) = 0.381, *p* = 0.537) (Figure 3A). When split based on the presence of VM in recurrent tumours, the VM− group (*n* = 25) had a longer median OS time of 553 days compared to the median OS time of 434 days for the VM+ recurrent group (*n* = 10). Again, the difference in OS distributions was not significant (χ^2^ (1) = 3.552, *p* = 0.060) (Figure 3B). However, there was a significant difference in post-progression survival (χ^2^ (1) = 4.830, *p* = 0.028), where the median survival of the VM− group (152 days; n = 20) was longer than that of the VM + group (92.5 days; *n* = 8) (Figure 3C). PFS distributions were not significantly different for VM+ vs. VM− tumour groups, regardless of whether VM was present in the primary or recurrent tumour (Supplementary Figure S1). 

### 3.5. Expression of PSMA Decreases at Recurrence

**Figure 4:**
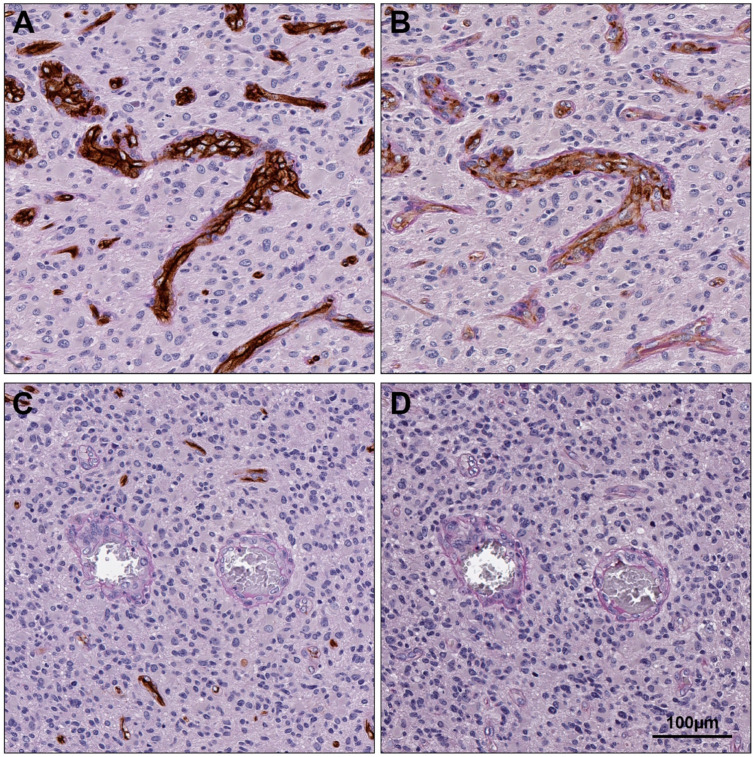
PSMA is expressed by endothelial vessels. Serial sections labelled by IHC for CD34 (**A**,**C**) and PSMA (**B**,**D**) demonstrate PSMA labelling in CD34+/PAS+ endothelial vessels, but not CD34−/PAS+ VM vessels. Scale bar = 100 µm.

**Figure 5:**
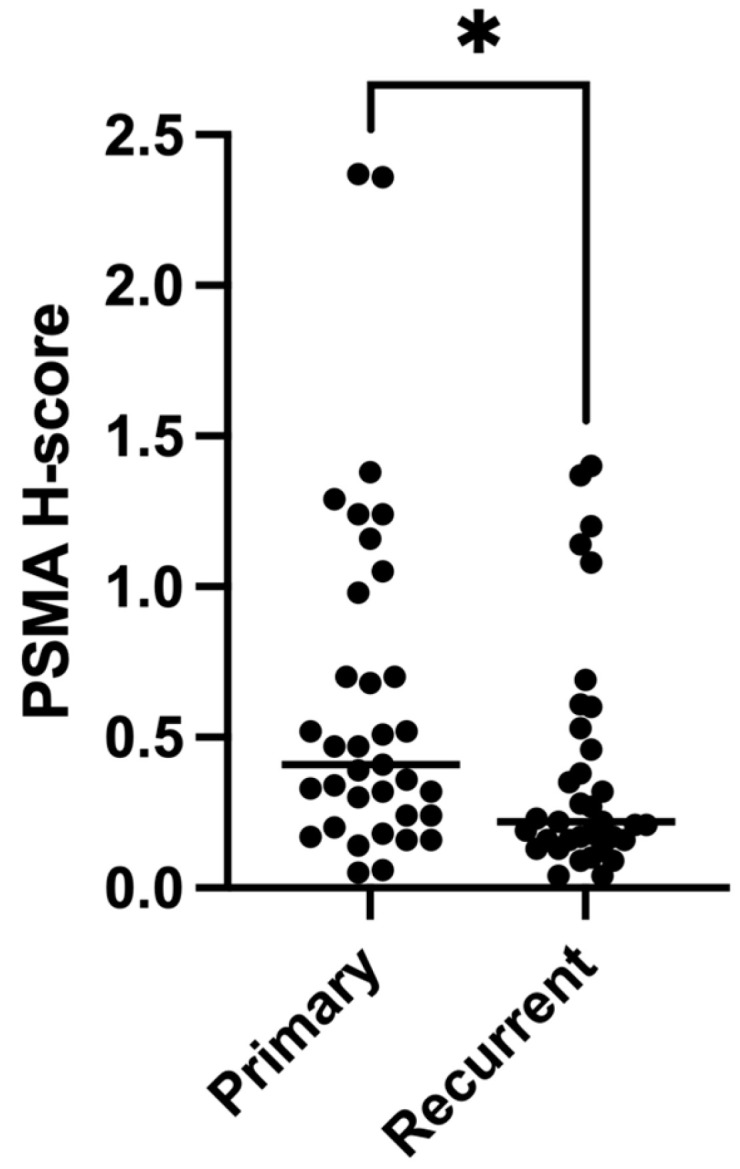
PSMA expression decreases at recurrence in glioblastoma. PSMA expression values (H-scores) were calculated based on percentage and intensity of tissue labelling by IHC as detected by HALO image analysis software. Expression of PSMA was significantly decreased in recurrent compared to primary glioblastoma (*z* = −1.925, one-tailed *p* = 0.027). Median values are shown for each dataset. * *p* < 0.05.

To determine which type(s) of vessels expressed PSMA in glioblastoma, serial sections of tissue were labelled by DAB IHC for PSMA or CD34 and stained using PAS. Vascular areas of tumour tissue that were PSMA+ were also CD34+ on the subsequent section, indicating that PSMA is expressed by endothelial vessels, not VM, in glioblastoma (Figure 4). We then tested whether, like endothelial vessel density, PSMA expression also decreased in recurrent tumours. There was a statistically significant decrease in PSMA expression in recurrent (H-score = 0.41) compared to primary (H-score = 0.22) glioblastoma (*z* = −1.925, one-tailed *p* = 0.027) (Figure 5). 

### 3.6. Lower PSMA Expression at Recurrence Is Associated with Shorter Post-Progression Survival

**Figure 6:**
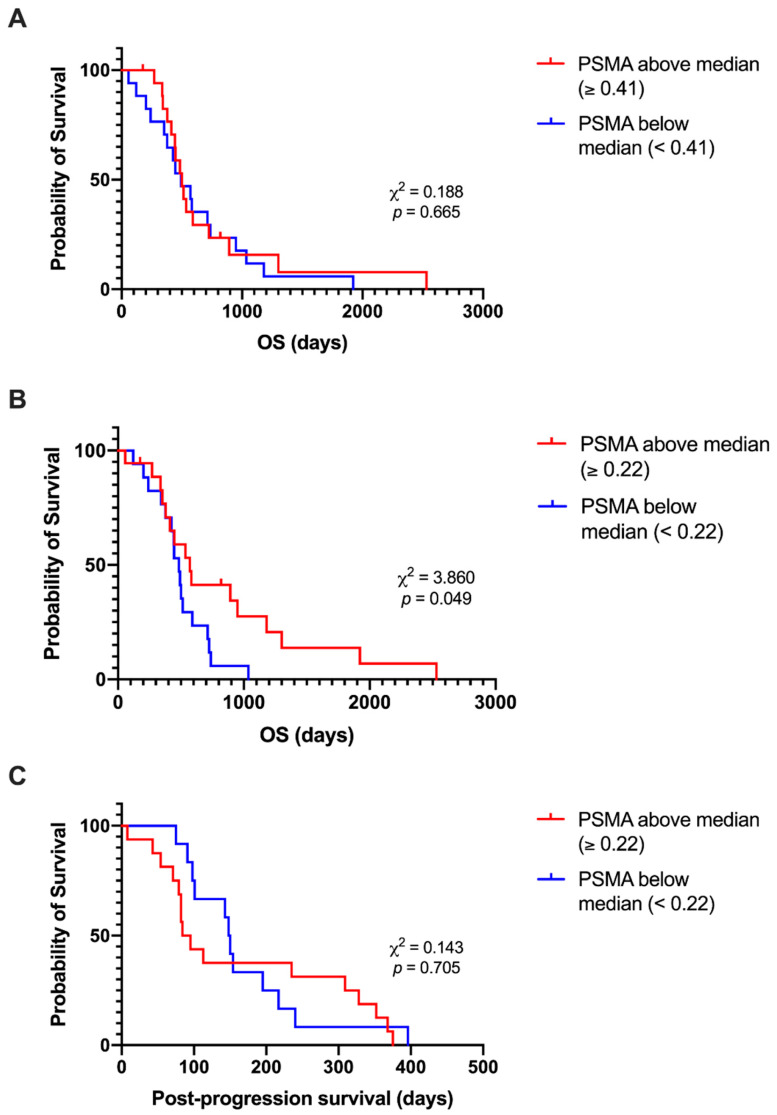
Kaplan–Meier plot of overall and post-progression survival distributions based on groupings of above- and below-median PSMA expression. Log rank tests determined that there was no significant difference in OS time for tumours with above-median PSMA expression compared to below-median PSMA expression in primary tumours (χ^2^ (1) = 0.188, *p* = 0.665) (**A**), but that cases with above-median PSMA expression at recurrence had longer OS time than those with below-median PSMA expression (χ^2^ (1) = 3.860, *p* = 0.049) (**B**). Post-progression survival time was not significantly different between groups (χ^2^ (1) = 0.143, *p* = 0.705) (**C**).

Kaplan–Meier survival analyses were performed as described above, with tumours grouped based on whether the PSMA expression was above or below the median value. Cases with above-median PSMA expression in the primary tumour (*n* = 18) had a median OS time of 501 days, which was not significantly different from the OS time of 493 days for the below-median PSMA group (*n* = 17; χ^2^ (1) = 0.188, *p* = 0.665) (Figure 6A). The median OS time for cases with above-median PSMA expression at recurrence (*n* = 18; 570 days) was longer than for cases with below-median PSMA expression (*n* = 17; 484 days; χ^2^ (1) = 3.860, *p* = 0.049) (Figure 6B). However, the post-progression survival distribution did not differ (χ^2^ (1) = 0.143, *p* = 0.705) (Figure 6C). PFS analyses were not statistically significant (Supplementary Figure S2). 

### 3.7. Relationships between Abnormal Vasculature and Clinical Characteristics

We next determined whether there were correlations between densities of any of the observed vessel types, in either primary or recurrent tumours, and clinical characteristics such as age at diagnosis and OS time. There was a statistically significant moderate positive correlation between VM vessel density in the primary tumour and age at diagnosis (τ_b_ = 0.343, *p* = 0.009). No other significant correlations between vessel densities and clinical features were observed (Supplementary Table S3). Interestingly, there was a weak-moderate negative correlation between VM and endothelial vessel densities at recurrence (τ_b_ = −0.273, *p* = 0.043). All correlations between densities of different vessel types are shown in Table 1. Despite our observation that PSMA is expressed by endothelial vessels in glioblastoma, and a significant decrease at recurrence in both CD34+ vessel density and PSMA expression, there was not a significant correlation between CD34+ vessel density and PSMA expression in either primary (τ_b_ = 0.175, *p* = 0.143) or recurrent (τ_b_ = 0.047, *p* = 0.691) glioblastoma. The complete correlation matrix is presented in Supplementary Table S3.

## 4. Discussion

It is now recognised that in addition to tumour angiogenesis, glioblastomas may develop vasculature through a number of alternative mechanisms including vessel co-option, vasculogenesis, tumour-cell-to-endothelial transdifferentiation, and vasculogenic mimicry (reviewed in [22,23]). In this study, we assessed characteristics of the tumour vasculature in matched patient samples of primary and recurrent glioblastoma tissue and report that VM is present in recurrent glioblastomas at a lower frequency than, but similar density to, primary tumours. We also observed a decreased expression of PSMA, a marker of pathological angiogenesis [15,19,24], in recurrent glioblastoma.

The reduction in overall vessel density at recurrence compared to primary glioblastoma was attributed to a significant decrease in the density of endothelial vessels, which accounted for most of the tumour vasculature. A decrease in CD34+ vessel density in recurrent glioblastoma has been previously reported [25,26], with one study suggesting that this is due to a reduction in total tumour tissue present after standard treatment [26]. Alternatively, decreased vessel density within the tumour may be the result of adaptive mechanisms, such as increased tumour cell invasion into the already vascularised surrounding tissue, similar to previous reports of increased tumour infiltration after bevacizumab treatment [4,27].

Labelling for the endothelial cell marker CD34 through immunohistochemistry (IHC) and staining with PAS is the most frequently used method of identifying VM vessels in glioblastoma tissue [28]. Using this method, we determined that 42.86% of primary tumours were VM+, which is within the previously reported range of 18–67% [11,12,13,14,20,29,30,31,32,33,34,35]. The majority of studies categorise tumours as either VM+ or VM−, but have used varying criteria when assigning tumours to each category, which may contribute to the variation in the proportion of VM+ tumours reported. For example, tumours may be considered VM+ if there are VM structures observed in any part of a whole tissue section [12,36], if VM structures are observed in randomly selected areas or regions of interest [13,34], or only if there are more than a certain number of VM structures observed [32,37]. In addition to categorising each tumour, we also quantified the mean VM vessel density of the tumours, demonstrating that the density of VM vessels did not change between primary and recurrent tumours and that VM accounts for only a very small proportion of the overall vasculature in glioblastoma.

Although we report a smaller proportion of VM vessels, our results are in agreement with those of a previous study, which also demonstrated that VM vessels represent a minor component of the glioblastoma vasculature [13]. Liu et al. previously reported an association between the categorical presence of VM and lower CD34+ vessel density in grade III and IV gliomas, and suggested that VM could be compensating for reduced endothelial vessel density in order to increase the tumour blood supply [12]. While we did not observe a significant relationship between density of CD34+ vessels and VM in primary glioblastoma, we did see a negative correlation between these vessel types in recurrent tumours, though this is potentially due to the reduction in endothelial vessel density at recurrence rather than the compensatory activity of tumour-derived vessels, as the correlation was relatively weak and the VM vessel density did not change significantly from primary tumours to recurrence. 

Despite VM making a minor contribution to the overall vascular density in glioblastoma, a shorter overall survival time has been reported for glioblastoma patients with VM+ primary tumours [13,14]. We also observed a shorter median overall survival time in VM+ compared to VM− primary tumours, though the difference in survival distributions was not statistically significant in our cohort. However, the post-progression survival time was significantly different with VM− cases having a longer survival time after recurrent tumour resection compared to VM+ cases. Further investigations are required to confirm that these survival differences are due to the presence of VM as opposed to other clinical factors. This is a limitation of the retrospective design of our study as some demographic, clinical, and treatment information was not available for all cases in our cohort. A prospective study where cases are selected based on the current WHO classification of glioblastoma would enable molecular and clinical features to be corrected for in a more in-depth analysis of the prognostic significance of VM. 

In addition to completely CD34− vessels, we observed vessels that were partially CD34+ in a subset of both primary and recurrent glioblastomas. We categorised these as “mosaic” vessels, consistent with prior reports of these vessels in glioblastoma [13,20]. One study that quantified mosaic vessels in primary glioblastomas observed that this vessel type occurred to a lesser extent than VM [13]. In contrast, we observed that there was a slightly higher density of mosaic vessels than VM vessels in both the primary and recurrent tumour groups. It has been suggested that mosaic vessels represent an intermediate stage of tumour-derived vessel development between VM and the complete differentiation of glioblastoma cells into functional endothelial cells [13], similar to the process of tumour to endothelial cell transdifferentiation. Alternatively, El Hallani et al. suggested that mosaic vessels are the result of tumour cell invasion into the blood vessel wall [20]. The loss of endothelial cells from the vessel lining, with or without tumour cell invasion, and the subsequent exposure of tumour cells to the vessel lumen have also been suggested as the mechanism of mosaic vessel formation in a study of colon carcinoma xenografts [38]. The use of additional endothelial and tumour cell markers in the assessment of glioblastoma tissue is required to determine which of these processes may be occurring. 

PSMA is expressed by the vasculature of numerous tumour types. In agreement with our results, the expression of PSMA in endothelial-cell-lined vessels in glioblastoma has been previously reported [18,26]. In this study, we report a decrease in PSMA expression at recurrence in glioblastoma, which is likely a reflection of the decreased density of endothelial vessels. This would support the results of a previous study, which showed a reduction in the number of both CD34+ and PSMA+ vessels in recurrent compared to matched primary glioblastoma samples [26]. The same study reported a longer median post-recurrence survival time for cases with below-median vascular PSMA expression, though this was not statistically significant in their cohort of 16 cases [26]. Another study reported that vascular PSMA expression was associated with shorter overall survival time in glioblastoma, while non-vascular PSMA expression was not [18]. Due to the automatic method of detecting and quantifying PSMA in our cohort, we were unable to distinguish vascular and non-vascular PSMA expression, which may contribute to our observations of no significant post-progression survival differences based on recurrent PSMA expression, and no overall survival differences based on primary PSMA expression, conflicting with those of prior studies. We did note a shorter overall survival for cases with below-median PSMA expression at recurrence, which may suggest that additional tumour or microenvironmental factors are affecting survival time in cases where tumour angiogenesis is reduced. 

While we did not observe PSMA+ VM structures in our glioblastoma cases, the possibility of PSMA expression by glioblastoma-derived vasculature cannot be entirely excluded. PSMA is expressed not only by vessels within tumours, but also by a small proportion of glioblastoma cells, which, in addition to VM, may also undergo the process of endothelial transdifferentiation in order to contribute to vessel formation [39,40]. In neuroblastoma, tumour-derived endothelial cells have been shown to express PSMA in addition to other typical endothelial cell markers such as CD31 and VE-cadherin [41]. While several studies have investigated the endothelial markers expressed by glioblastoma-derived endothelial cells [39,40,42,43,44,45,46], none of these assessed the expression of PSMA. Whether PSMA is expressed by glioblastoma cells in the context of endothelial transdifferentiation requires further investigation.

## 5. Conclusions

Changes that occur after treatment in recurrent glioblastoma extend to aspects of the tumour microenvironment, including the tumour vasculature. These changes include a decrease in endothelial vessel density and decreased expression of PSMA, suggesting a reduced dependence on tumour angiogenesis. Categorical assessment of VM presence at recurrence suggested an association between VM and shorter post-progression survival. However, VM accounted for a minor proportion of the tumour vasculature in both the primary and recurrent setting, and the extent to which VM makes a functional contribution to the overall vasculature requires further study in order to determine whether VM is a relevant treatment target.

## Figures and Tables

**Table 1:** Vessel density correlations in primary and recurrent glioblastoma.

		Endothelial Vessel Density, Primary	Endothelial Vessel Density, Recurrent	VM Vessel Density, Primary	VM Vessel Density, Recurrent	Mosaic Vessel Density, Primary	Mosaic Vessel Density, Recurrent	Total Vessel Density, Primary	Total Vessel Density, Recurrent
**Endothelial Vessel Density, Primary**	**τ_b_**	−							
	***p*-value**	−							
**Endothelial Vessel Density, Recurrent**	**τ_b_**	0.135	−						
	***p*-value**	0.256	−						
**VM Vessel Density, Primary**	**τ_b_**	0.006	0.100	−					
	***p*-value**	0.962	0.447	−					
**VM Vessel Density, Recurrent**	**τ_b_**	0.024	−0.273 *	−0.009	−				
	***p*-value**	0.858	0.043	0.952	−				
**Mosaic Vessel Density, Primary**	**τ_b_**	−0.029	−0.017	0.401 **	0.082	−			
	***p*-value**	0.808	0.886	0.003	0.553	−			
**Mosaic Vessel Density, Recurrent**	**τ_b_**	0.028	−0.095	0.163	0.483 **	0.257 *	−		
	***p*-value**	0.819	0.439	0.231	<0.001	0.041	−		
**Total Vessel Density, Primary**	**τ_b_**	0.974 **	0.147	0.033	0.012	0.000	0.016	−	
	***p*-value**	<0.001	0.216	0.800	0.929	1.000	0.897	−	
**Total Vessel Density, Recurrent**	**τ_b_**	0.133	0.908 **	0.131	−0.189	0.019	0.005	0.145	−
	***p*-value**	0.262	<0.001	0.318	0.162	0.875	0.966	0.222	−

** Correlation is significant at the 0.01 level; * Correlation is significant at the 0.05 level; All *p*-values are two-tailed.

## Data Availability

The data presented in this study are available on reasonable request from the corresponding author.

## Supplementary Materials

The following supporting information can be downloaded at https://www.mdpi.com/article/10.3390/cancers15153922/s1, Table S1: Patient demographic and clinical characteristics; Table S2: Mean density of tumour vessels in each vessel category, and total vessel density, in primary and recurrent glioblastoma groups; Figure S1: Progression-free survival did not differ between VM+ and VM− glioblastoma; Figure S2: Progression-free survival did not differ between above- and below-median expression of PSMA in glioblastoma; Table S3: Correlations between vessel densities and clinical characteristics.
