# Supplementary material for: Vasculogenic Mimicry Occurs at Low Levels in Primary and Recurrent Glioblastoma
Source: Cancers (Basel). 2023 Aug 1;15(15):3922. doi: 10.3390/cancers15153922 (PMC10417556; doi:10.3390/cancers15153922)
Supplement: Supplementary file 1 [file cancers-15-03922-s001.zip › cancers-2519026-supplementary.pdf]

Supplementary Materials: Vasculogenic Mimicry Occurs at Low Levels in Primary and Recurrent Glioblastoma

Kelsey Maddison, Sam Faulkner, Moira C. Graves, Michael Fay, Nikola A. Bowden and Paul A. Tooney

Table S1. Patient demographic and clinical characteristics.

**Characteristic**		***n***	**%**
Median age at diagnosis, years (range)	59 (23–81)		
Sex			
Male		20	57.14
Female		15	42.86
Treatment after primary resection			
Radiation		1	2.86
TMZ		0	0.00
Radiation + TMZ		29	82.86
None		2	5.71
Unknown		3	8.57
IDH1 mutation status			
WT		23	65.71
Mutant		1	2.86
Unknown		11	31.43
MGMT promoter methylation status			
Methylated		4	11.43
Unmethylated		6	17.14
Unknown		25	71.43
Recurrent tissue collection timepoint			
Surgical resection		30	85.71
Post-mortem		5	14.29
Median overall survival, days (range)	493 (57–2531)		

Note *IDH* mutation status and *MGMT* methylation status were not available for all cases. Treatment information was unavailable or incomplete for some cases.

Table S2. Mean density of tumour vessels in each vessel category, and total vessel density, in primary and recurrent glioblastoma groups.

	**Vessel Density (Vessels/mm²; Mean ± SD)**			
	**Endothelial Vessels**	**VM**	**Mosaic Vessels**	**Total Vessels**
**Primary**	87.79 ± 47.07	0.58 ± 1.16	2.33 ± 2.40	90.70 ± 46.87
**Recurrent**	51.91 ± 39.09	0.37 ± 1.10	2.10 ± 2.69	54.38 ± 38.54

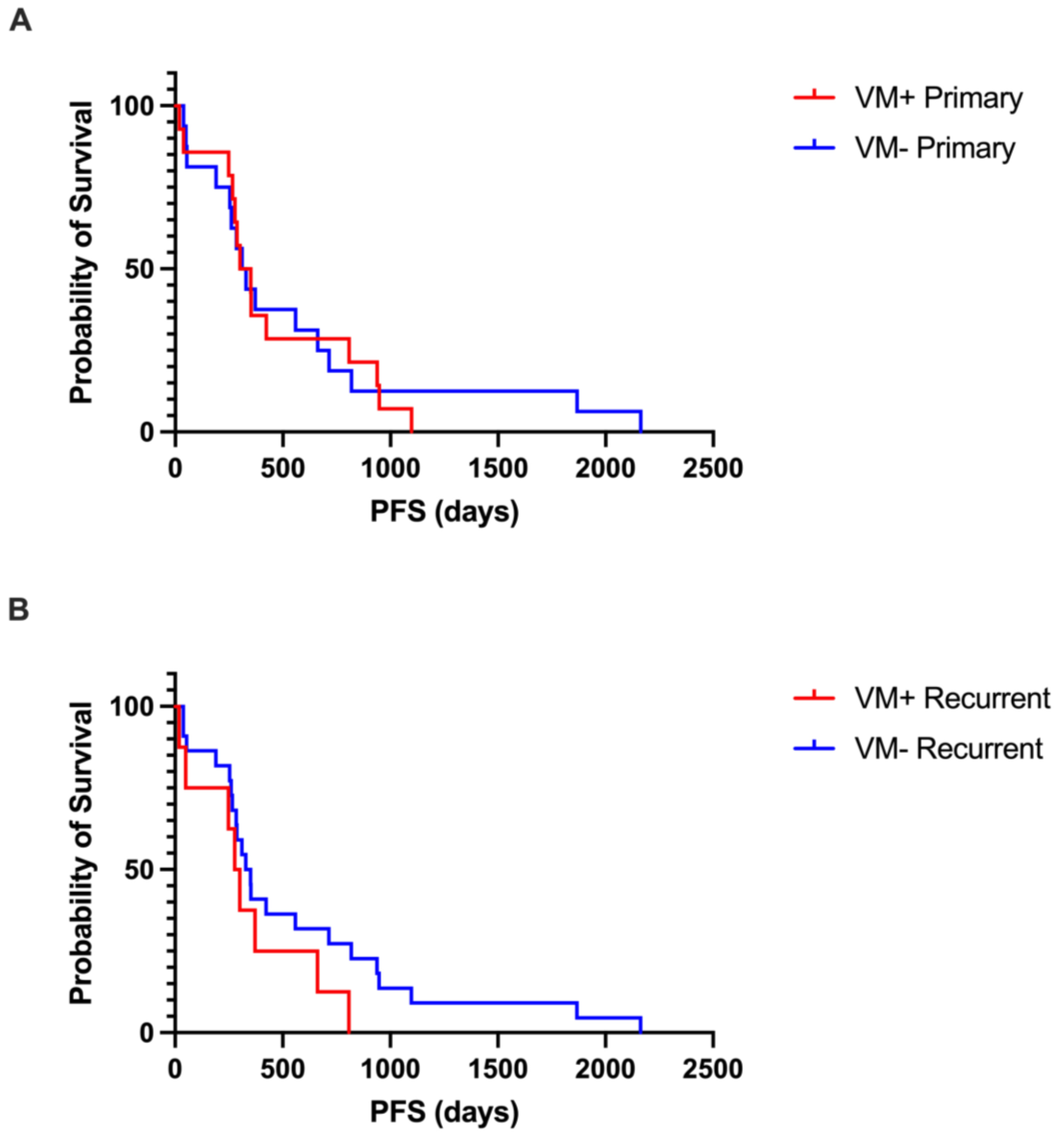

Figure S1. Progression-free survival did not differ between VM+ and VM– glioblastoma. Kaplan-Meier survival analyses with log rank tests were used to assess differences in progression-free survival (PFS) between VM+ and VM– groups. The median PFS time was similar when tumours were grouped based on whether VM occurred in the primary tumour, with the VM+ group ($n = 14$) having a median PFS of 325 days and the VM– group ($n = 16$) having a PFS time of 319 days ($\chi^2(1) = 0.075$, $p = 0.784$). When grouped based on VM in the recurrent tumour, the VM+ cases ($n = 8$) had shorter median PFS (288.5 days) than the VM– group ($n = 22$; median PFS 339 days), but this was not statistically significant ($\chi^2(1) = 1.807$, $p = 0.179$). PFS analyses were performed for $n = 30$ cases; 5 cases for which recurrent tissue was collected post-mortem were excluded.

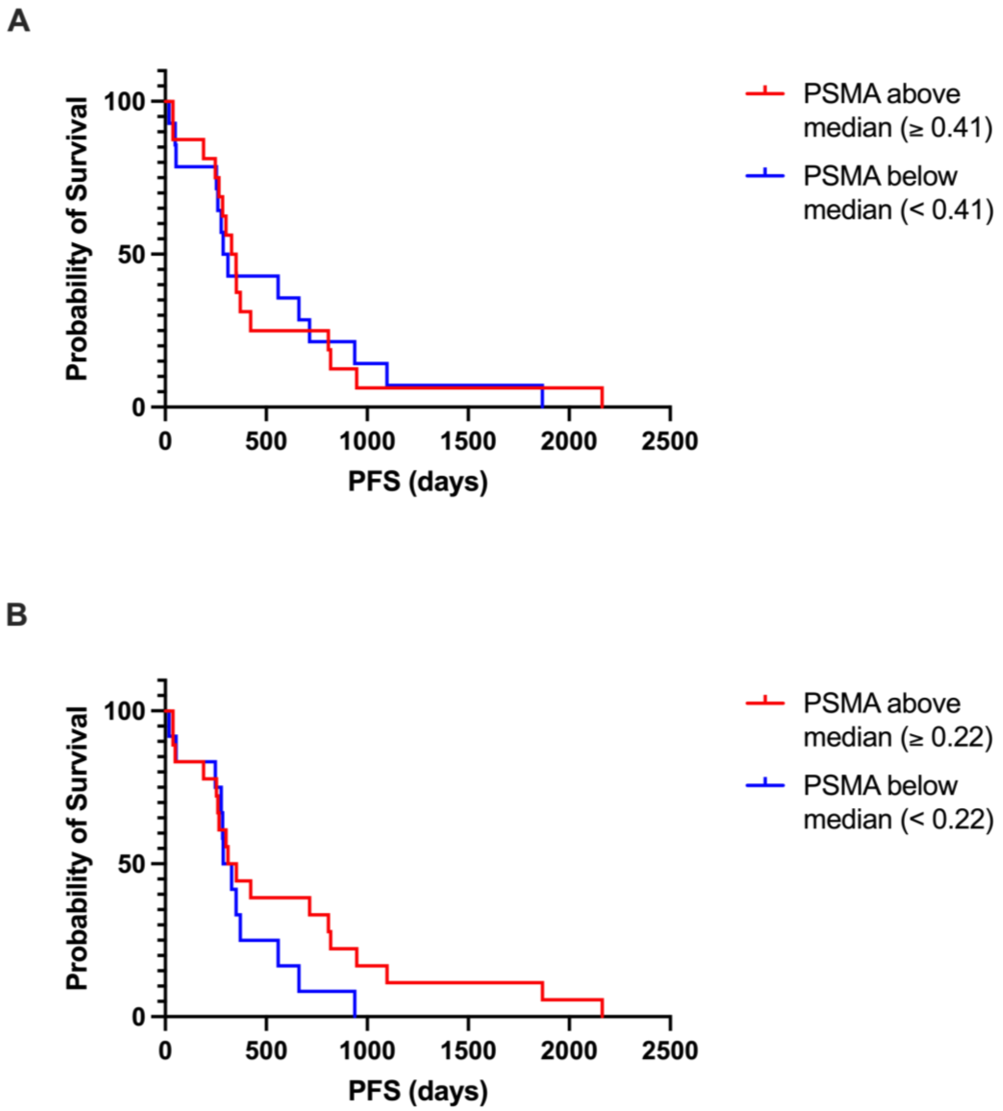

Figure S2. Progression-free survival did not differ between above- and below-median expression of PSMA in glioblastoma. Progression-free survival (PFS) was analysed using the Kaplan-Meier method with log rank tests. Based on PSMA expression in primary tumours, there was not a statistically significant difference ($\chi^2(1) < 0.001$, $p = 0.975$) in the median PFS of tumours with above-median PSMA expression ($n = 16$; PFS 339 days) and below-median PSMA expression ($n = 14$; PFS 298.5 days). The median PFS for cases with above median PSMA expression at recurrence ($n = 18$) was 331 days, which was not significantly different from the PFS of 307.5 days for the below-median PSMA expression group ($n = 12$; $\chi^2(1) = 1.773$, $p = 0.183$). PFS analyses were performed for $n = 30$ cases; 5 cases for which recurrent tissue was collected post-mortem were excluded.

Table S3. Correlations between vessel densities and clinical characteristics.

		Age at Diagnosis	Overall Survival	Progression-Free Survival	Post-Progression Survival	Endothelial Vessel Density, Primary	Endothelial Vessel Density, Recurrent	VM Vessel Density, Primary	VM Vessel Density, Recurrent	Mosaic Vessel Density, Primary	Mosaic Vessel Density, Recurrent	Total Vessel Density, Primary	Total Vessel Density, Recurrent	PSMA H-Score, Primary	PSMA H-Score, Recurrent
**Age at Diagnosis**	τ_b	–													
	p-value	–													
	n	–													
**Overall Survival**	τ_b	−0.053	–												
	p-value	0.659	–												
	n	35	–												
**Progression-Free Survival**	τ_b	−0.025	0.750**	–											
	p-value	0.844	<0.001	–											
	n	30	30	–											
**Post-Progression Survival**	τ_b	−0.219	0.241	−0.029	–										
	p-value	0.105	0.072	0.828	–										
	n	28	28	28	–										
**Endothelial Vessel Density, Primary**	τ_b	−0.058	−0.003	−0.078	0.098	–									
	p-value	0.629	0.977	0.544	0.465	–									
	n	35	35	30	28	–									
**Endothelial Vessel Density, Recurrent**	τ_b	0.053	0.126	0.051	−0.032	0.135	–								
	p-value	0.659	0.287	0.695	0.813	0.256	–								
	n	35	35	30	28	35	–								
**VM Vessel Density, Primary**	τ_b	0.343**	0.056	0.091	−0.071	0.006	0.100	–							
	p-value	0.009	0.669	0.520	0.625	0.962	0.447	–							
	n	35	35	30	28	35	35	–							
**VM Vessel Density, Recurrent**	τ_b	0.198	−0.193	−0.140	−0.264	0.024	−0.273*	−0.009	–						
	p-value	0.146	0.152	0.344	0.085	0.858	0.043	0.952	–						
	n	35	35	30	28	35	35	35	–						
**Mosaic Vessel Density, Primary**	τ_b	0.140	−0.048	−0.019	−0.202	−0.029	−0.017	0.401**	0.082	–					
	p-value	0.253	0.689	0.886	0.142	0.808	0.886	0.003	0.553	–					
	n	35	35	30	28	35	35	35	35	–					
**Mosaic Vessel Density, Recurrent**	τ_b	0.151	−0.148	−0.075	−0.268	0.028	−0.095	0.163	0.483**	0.257*	–				
	p-value	0.222	0.229	0.577	0.053	0.819	0.439	0.231	<0.001	0.041	–				
	n	35	35	30	28	35	35	35	35	35	–				
**Total Vessel Density, Primary**	τ_b	−0.060	0.008	−0.078	0.087	0.974**	0.147	0.033	0.012	0.000	0.016	–			
	p-value	0.619	0.943	0.544	0.514	<0.001	0.216	0.800	0.929	1.000	0.897	–			
	n	35	35	30	28	35	35	35	35	35	35	–			
**Total Vessel Density, Recurrent**	τ_b	0.095	0.098	0.032	−0.045	0.133	0.908**	0.131	−0.189	0.019	0.005	0.145	–		
	p-value	0.426	0.410	0.803	0.737	0.262	<0.001	0.318	0.162	0.875	0.966	0.222	–		
	n	35	35	30	28	35	35	35	35	35	35	35	–		
**PSMA H-Score, Primary**	τ_b	−0.073	0.020	0.023	0.224	0.175	−0.068	0.006	−0.032	−0.033	0.119	0.156	−0.083	–	
	p-value	0.541	0.865	0.858	0.097	0.143	0.570	0.962	0.816	0.786	0.337	0.191	0.486	–	
	n	35	35	30	28	35	35	35	35	35	35	35	35	–	
**PSMA H-Score, Recurrent**	τ_b	0.041	0.169	0.107	−0.104	−0.100	0.047	0.136	−0.170	0.150	−0.035	−0.088	0.025	0.022	–
	p-value	0.733	0.155	0.411	0.440	0.401	0.691	0.303	0.210	0.219	0.774	0.460	0.831	0.853	–
	n	35	35	30	28	35	35	35	35	35	35	35	35	35	–

** Correlation is significant at the 0.01 level; * Correlation is significant at the 0.05 level; All *p*-values are two-tailed.
